# Toll-like receptor 2 activation and serum amyloid A regulate smooth muscle cell extracellular matrix

**DOI:** 10.1371/journal.pone.0171711

**Published:** 2017-03-03

**Authors:** Stephanie E. Seidl, Lawrence G. Pessolano, Christopher A. Bishop, Michael Best, Celeste B. Rich, Phillip J. Stone, Barbara M. Schreiber

**Affiliations:** Department of Biochemistry, Boston University School of Medicine, Boston, Massachusetts, United States of America; University of Insubria, ITALY

## Abstract

Smooth muscle cells contribute to extracellular matrix remodeling during atherogenesis. De-differentiated, synthetic smooth muscle cells are involved in processes of migration, proliferation and changes in expression of extracellular matrix components, all of which contribute to loss of homeostasis accompanying atherogenesis. Elevated levels of acute phase proteins, including serum amyloid A (SAA), are associated with an increased risk for atherosclerosis. Although infection with periodontal and respiratory pathogens via activation of inflammatory cell Toll-like receptor (TLR)2 has been linked to vascular disease, little is known about smooth muscle cell TLR2 in atherosclerosis. This study addresses the role of SAA and TLR2 activation on smooth muscle cell matrix gene expression and insoluble elastin accumulation. Cultured rat aortic smooth muscle cells were treated with SAA or TLR2 agonists and the effect on expression of matrix metallopeptidase 9 (MMP9) and tropoelastin studied. SAA up-regulated MMP9 expression. Tropoelastin is an MMP9 substrate and decreased tropoelastin levels in SAA-treated cells supported the concept of extracellular matrix remodeling. Interestingly, SAA-induced down-regulation of tropoelastin was not only evident at the protein level but at the level of gene transcription as well. Contributions of proteasomes, nuclear factor κ B and CCAAT/enhancer binding protein β on regulation of MMP9 vs. tropoleastin expression were revealed. Effects on *Mmp9* and *Eln* mRNA expression persisted with long-term SAA treatment, resulting in decreased insoluble elastin accumulation. Interestingly, the SAA effects were TLR2-dependent and TLR2 activation by bacterial ligands also induced MMP9 expression and decreased tropoelastin expression. These data reveal a novel mechanism whereby SAA and/or infection induce changes in vascular elastin consistent with atherosclerosis.

## Introduction

Atherosclerosis involves chronic vascular inflammation with evidence for involvement of innate and adaptive arms of the immune system [1]. Smooth muscle cells (SMCs) contribute to inflammation and extracellular matrix remodeling. SMCs are not terminally differentiated and the phenotypic switch from a contractile cell is involved in processes of migration, proliferation and changes in expression of extracellular matrix components, all of which contribute to the loss of homeostasis accompanying atherogenesis [2].

Elevated plasma levels of acute phase proteins, including liver-derived serum amyloid A (SAA), are associated with an increased risk for atherosclerosis. Both pro-atherogenic and anti-atherogenic functions have been attributed to the SAA family [3, 4]. Macrophage-synthesized SAA accelerates early lesion development [5]. Of potential importance, SAA up-regulates matrix metallopeptidase (MMP)9 expression in THP-1 monocytes and synovial explants from arthritis patients [6, 7]. Our laboratory and others showed that SMCs also express SAA [8, 9].

A number of SAA receptors have been demonstrated, including Toll-like receptor (TLR)2 [10–12] and TLR4 [13], although the structure of SAA differs from ligands classically associated with these receptors. Activation of TLR2 and TLR4 has been linked to inflammation and atherosclerosis [14] *e*.*g*. TLR2 activation induces monocyte/macrophage MMP9 expression [15, 16] but little is known about SMC TLR2 in atherosclerosis. The hypothesis that SAA decreases SMC elastin accumulation was tested. SAA up-regulated MMP9 expression and down-regulated tropoelastin expression, resulting in decreased insoluble elastin accumulation. The effects were TLR2-dependent and TLR2 activation by bacterial ligands also induced the gene expression changes. With the known association between SAA and atherosclerosis, this study offers a mechanism for disease progression. Moreover, the data link TLR2 activation by infection with periodontal pathogens *e*.*g*. *P*. *gingivalis* as well as respiratory pathogens *e*.*g*. *Chlamydia pneumonia* and changes in vascular extracellular matrix consistent with atherosclerosis [17, 18].

## Materials and methods

### SMC cultures and experimental design

**Fig 1:**
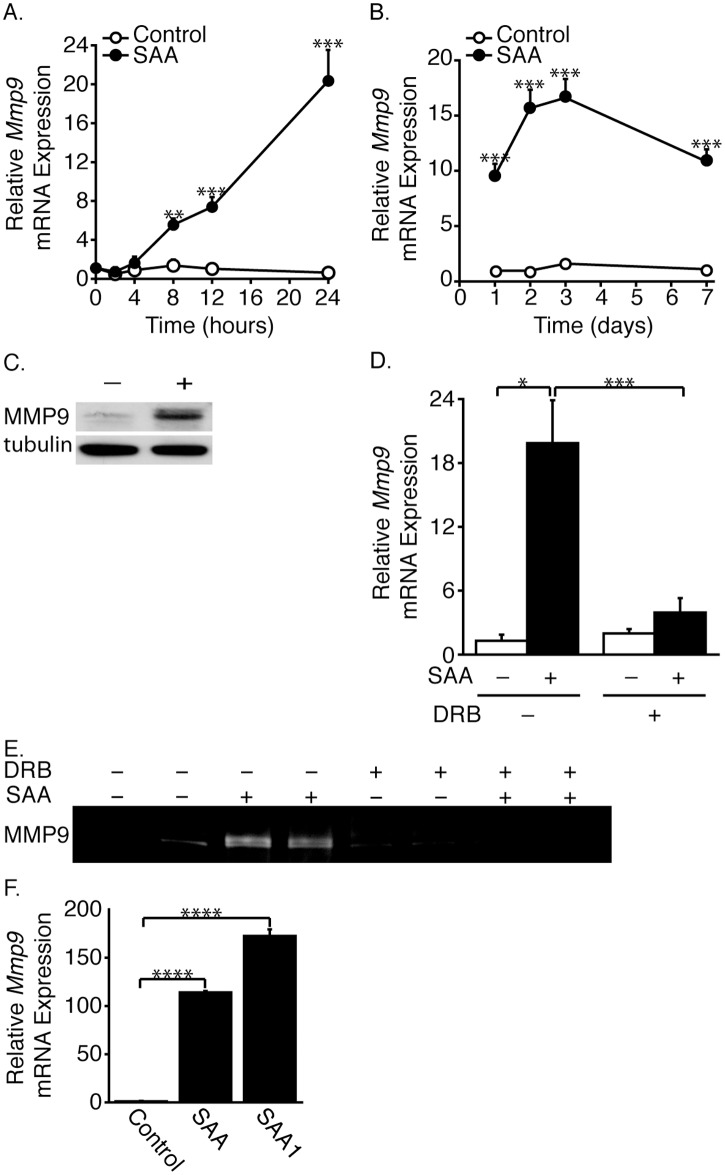
SAA increases MMP9 expression. SMCs were treated (or control-treated) with SAA for the indicated time (A, B). *Mmp9* mRNA levels are expressed relative to the 0-hour control-treated (A) or 1-day control-treated (B) sample ± SD (n = 3). SMCs were treated (or control-treated) with SAA for 24 hours and Western blot analysis performed with antibodies directed against MMP9 and tubulin (C). SMCs were pretreated (or control-treated) with DRB, then treated (or control-treated) with SAA and incubated for 16 hours (D, E). *Mmp9* mRNA levels are expressed relative to the control-treated (no SAA or DRB) sample ± SD (n = 3) (D). Media were harvested and subjected to zymography (E). SMCs were treated with SAA or SAA1 as in Fig 1C (F). *Mmp9* mRNA levels are expressed relative to the control-treated sample ± SD (n = 3).

In accordance with practices approved by the Institutional Animal Care and Use Committee at Boston University, 3-day old Sprague-Dawley rats (Charles River Laboratories, Wilmington, MA) were euthanized for cell isolation on day of arrival (Protocol AN-14307). The neonates were euthanized by decapitation; the dams (delivered with the neonates but not used for cell isolation) were euthanized by CO_2_ inhalation followed by decapitation. SMCs were isolated by elastase and collagenase digestion of aortas as previously described [19]. Experiments were performed on confluent cells in first or second passage and seeded in Dulbecco’s Modified Eagle’s Medium (Cellgro by Mediatech, Manasses, VA) containing 100 IU/ml penicillin, 100 μg/ml streptomycin (Cellgro), 1 μM sodium pyruvate (Cellgro), 1 μM non-essential amino acids (DMEM; Cellgro) and 10% fetal bovine serum (Atlanta Biologicals, Lawrenceville, GA). Lipoprotein deficient serum was prepared as previously described [20]. Prior to SMC treatment, media were removed and cells were washed twice with Hank’s Balanced Salt Solution (HBSS; Cellgro). DMEM with 10% lipoprotein deficient serum was added, followed by treatment with agonists and/or inhibitors. To mirror conditions used for the zymogram in Fig 1E, mRNA expression shown in Fig 1D was performed on cells treated in media containing 0.5% lipoprotein deficient serum but this was confirmed using 10% lipid deficient serum as for all other studies (data not shown).

Reagents included two recombinant human SAA preparations; 1) the preparation referred to as SAA is a hybrid that corresponds to human SAA1α, except for an N-terminal methionine and 2 amino acid substitutions found in SAA2β (an asparagine substituted for aspartic acid at position 60, and an arginine for histidine at position 71; 2 μM unless otherwise indicated) and 2) the preparation referred to as SAA1 corresponds to human SAA1α except for an N-terminal methionine (2 μM) both from PeproTech, Rocky Hill, NJ, 5,6-dichloro-1-β-D-ribofuranosylbenzimidazole (DRB; 20 μg/ml; Sigma-Aldrich Co., St. Louis, MO), MG-132 (10 μM; Invitrogen/Life Technologies, Grand Island, NY), N-tosyl-L-phenylalanine chloromethyl ketone (TPCK; 10, 25 μM; Sigma-Aldrich Co.), high density lipoprotein (HDL; 150 μg protein/ml; Calbiochem, La Jolla, CA), Congo red (Sigma-Aldrich Co.), interleukin-1 (IL-1) receptor (IL-1R) antagonist (IL-1Ra; 1 μg/ml; R&D Systems, Minneapolis, MN), IL-1β (100 ng/ml; eBioscience Inc., San Diego, CA), polymyxin B (PxB; 10 μg/ml; InvivoGen, San Diego, CA), CLI-095 (TAK; 1 μg/ml; InvivoGen) and TLR agonists including lipopolysaccharide from *Escherichia coli* K12 (*E*. *coli* LPS; 100 ng/ml), lipopolysaccharide from Gram-negative *Porphyromonas gingivalis* (*Pg* LPS; 1 μg/ml), lipoteichoic acid from Gram-positive *Staphylococcus aureus* (LTA; 1 μg/ml), synthetic triacylated lipoprotein, Pam3CSK4 (Pam; 1 μg/ml) and synthetic diacylated lipoprotein, FSL-1 (FSL; 1 μg/ml) all from InvivoGen. For inhibitor studies, cells were pretreated for 1 hour before the addition of SAA or other agonists. Media and reagents were replaced twice weekly for long-term studies (greater than 48 hours) and always the day before cells were harvested.

### QPCR

QPCR was performed as previously described [21]. Essentially, total RNA was extracted and subjected to reverse transcriptase polymerase chain reaction to generate cDNA that was used for the analyses. SYBR and TaqMan primers for detection of levels of mRNA are listed in Tables 1 and 2, respectively. To detect *Eln* heteronuclear (hn)RNA, the following primers were designed using Primer Express Software (Applied Biosystems): forward 5’ ACCTCATCCTCTGCCAACAC, reverse 5’ GCTGGTGGACCTAGACCTTG and Taq probe 5’ AAAACCCCCGAAGCCCTA. All data were normalized against the expression of 18S rRNA. For each experiment, one control value was chosen as the standard to which the other control samples as well as all experimental values were compared. The data, expressed as mRNA or hnRNA expression ± SD, represent fold change relative to the control value to which all other samples (including other controls) were compared.

**Table 1:** SYBR qPCR rat primers.

Gene	Source
*Cebpb*	Invitrogen NCBI NM_024125.4
*Cxcl1*	[22]
*Mmp9*	Invitrogen NCBI NM_031055.1
*Nos2*	[23]

**Table 2:** TaqMan qPCR primers.

Gene	Species	Applied Biosystems TaqMan Assay ID
*18S rRNA*	Human	Hs99999901_s1
*Col1a1*	Rat	Rn00801649_g1
*Eln*	Rat	Rn01499782_m1
*Tlr2*	Rat	Rn02133647_s1

### Western blot analysis

Western blot analysis was performed as previously described [21]. Essentially, after cells were lysed [1% Triton X-100, 0.15 M NaCl, 0.01 M Tris pH 7.5, 1 mM EDTA pH 7.5, 1 mM EGTA pH 9.0, 0.5% NP-40, 0.4 mM phenylmethylsulfonyl fluoride and 0.2 mM sodium vanadate], diisofluorophosphate was added to a final concentration of 1 μM, lysates were kept on ice for 30 minutes and stored at -80°C. The total protein concentration was determined using the BCA Protein Assay Kit (Pierce, Rockford, IL) and 10 μg were loaded in each lane. Primary antibodies included goat anti-rat tropoelastin [RA-75; Elastin Products Company, Inc., Owensville, MO], rabbit anti-rat MMP9 catalytic domain (Chemicon EMD Millipore Corporation, Temecula, CA) or anti-mouse α-tubulin clone DM1A (loading control; Sigma-Aldrich Co.). Horseradish peroxidase-conjugated secondary antibodies were from Santa Cruz Biotechnology (Santa Cruz, CA). Signal was detected with the Enhanced Chemiluminescence Western Blotting Substrate (Pierce).

### Transient transfections and luciferase assays

Transient transfection analysis was performed as previously described [21]. Constructs containing 216 bp of the rat elastin promoter driving luciferase, referred to as 216-luc [24] and a nuclear factor κ B (NFκB) reporter construct driving luciferase expression, referred to as NFκB-luc [21] were used. Cultures were co-transfected with the experimental construct and a pRL-CMV-*Renilla* construct (Promega, Madison, WI) (the latter for normalization of transfection efficiency). To achieve optimal efficiency, transfections were performed using FuGene 6 (Roche, Indianapolis; following the manufacturer’s instructions) when SMCs were approximately 70% confluent. Twenty-four hours after transfection, media were removed and the cells were cultured under experimental conditions as indicated. At the time of harvest, media were removed, cells were washed twice with cold HBSS and placed at -80°C for at least 24 hours. To determine luciferase and *Renilla* activities, the Dual-luciferase Reporter Assay (Promega) kit was used as per manufacturer’s instructions. Data are expressed as luciferase/*Renilla* ± SD.

### Zymography

MMP activity was detected by gelatin zymography. Media were removed and stored at -80°C. The conditioned media (5–15 μl/lane) were loaded onto a Novex^®^ Gelatin Zymogram (Invitrogen) with an equal volume of Novex Tris-Glycine SDS Sample Buffer (Invitrogen). Proteins were resolved by gel electrophoresis at 130 V for 2 hours. The gel was placed in Novex^®^ Zymogram Renaturing Buffer (Invitrogen) for 30 minutes followed by Novex^®^ Zymogram Developing Buffer (Invitrogen) for 30 minutes. The gel was transferred to fresh Novex^®^ Zymogram Developing Buffer and incubated at 37°C for 18 hours. The gel was rinsed in water and placed in SimplyBlue^™^ SafeStain (Invitrogen) on a rocker platform until clear bands representing MMP activity were visualized.

### Insoluble elastin determination by amino acid analysis

SMCs were cultured for 2 weeks before treatment with or without SAA for an additional 2 weeks, at which time, amino acid analysis was performed. Insoluble elastin was isolated from the cell layers using the procedure of Lansing et *al*. [25] and amino acid analysis performed to determine levels of insoluble elastin (hot alkali-insoluble protein) and all other protein (hot alkali-soluble fraction) essentially as previously described [26, 27]. The insoluble elastin was calculated as follows: the amount of glycine was multiplied by the mass of glycine (85 ng/nmol) and divided by the percent of glycine in insoluble elastin (33.7%). The protein in the supernatant *i*.*e*. hot alkali-soluble protein was calculated as follows: the amount of amino acids was multiplied by the average mass of amino acids (100 ng/nmol). The total protein in the sample was calculated as follows: hot alkali-insoluble (insoluble elastin) + hot alkali-soluble. The data are expressed as a function of the growing surface area *e*.*g*. insoluble elastin (μg/cm^2^ ± SD).

### Congo red analysis

Congo red staining of SMC insoluble elastin was performed as previously described [28]. Essentially, cells were cultured for 2 weeks before treatment with SAA for an additional 2 weeks, at which time, cultures were treated with 0.5 ml 0.1 N NaOH for 1 hour at 37°C. Cultures were washed 3 times with HBSS and incubated with Congo red (25 μg/ml) in 1 ml HBSS for 1 hour at 37°C. Cultures were again washed 3 times with HBSS and Congo red staining was observed by fluorescence.

### *Tlr2* gene silencing

SMCs were transfected with small interfering RNA targeting rat TLR2 (*siTlr2*; final concentration, 25 nM; siGenome SmartPool; Dharmacon, Lafayette, CO) or non-targeting control small interfering RNA (*siCtl*; Dharmacon). Cells at 70% confluence were washed twice with HBSS, DMEM without antibiotics containing 10% fetal bovine serum was added and transfection was performed using Lipofectamine RNAiMax (Invitrogen, Carlsbad, CA) according to the manufacturer’s instructions. After 24 hours, media were removed, cells were washed twice with HBSS and DMEM with 10% fetal bovine serum was added for an additional 24 hours, at which time, media were removed and the cells were cultured under experimental conditions as indicated.

### Statistics

All experiments were repeated at least 3 times with different batches of cells. Two-tailed unpaired Student’s t-test was used to compare 2 samples. Otherwise, ANOVA (one-way or two-way) was used to compare multiple samples and statistically significant differences of relevant comparisons were determined by Bonferroni post-hoc analysis. Statistically significant differences were reported when P<0.05, P < .01, P < .001, P < .0001 (indicated by *, **, ***, ****, respectively).

## Results

### SAA induces MMP9 expression

Involvement of the MMP family in extracellular matrix remodeling in atherosclerosis is documented [29] but the role of inflammation-induced MMPs on SMC elastin accumulation is unknown. *Mmp9* mRNA expression was monitored in SAA-treated neonatal rat aortic SMCs; expression increased as early as 8 hours after treatment (Fig 1A) and persisted with chronic treatment to 7 days (Fig 1B). A corresponding increase in MMP9 protein was evident (Fig 1C). The SAA-mediated *Mmp9* mRNA up-regulation was inhibited by DRB, an inhibitor of RNA polymerase II-mediated transcription [30], demonstrating that the effect was at the transcriptional level (Fig 1D). Gelatin zymography showed that bands consistent with active MMP9 were greater in conditioned media from SAA-treated *vs*. control-treated cultures and that this effect was lost in DRB-treated cells (Fig 1E).

The SAA hybrid molecule has been the subject of many studies but its physiological relevance has been questioned because of the amino acid substitutions, such that the sequence differs slightly from that of human SAA1α (see Materials and Methods section). Because of the concern regarding the use of the hybrid SAA, additional experiments were performed with recombinant human SAA1 (differing from SAA1α only by the N-terminal methionine). Importantly, the recombinant human SAA1 isoform was at least as effective in inducing *Mmp9* mRNA expression as the SAA hybrid molecule (Fig 1F).

### SAA decreases tropoelastin expression

**Fig 2:**
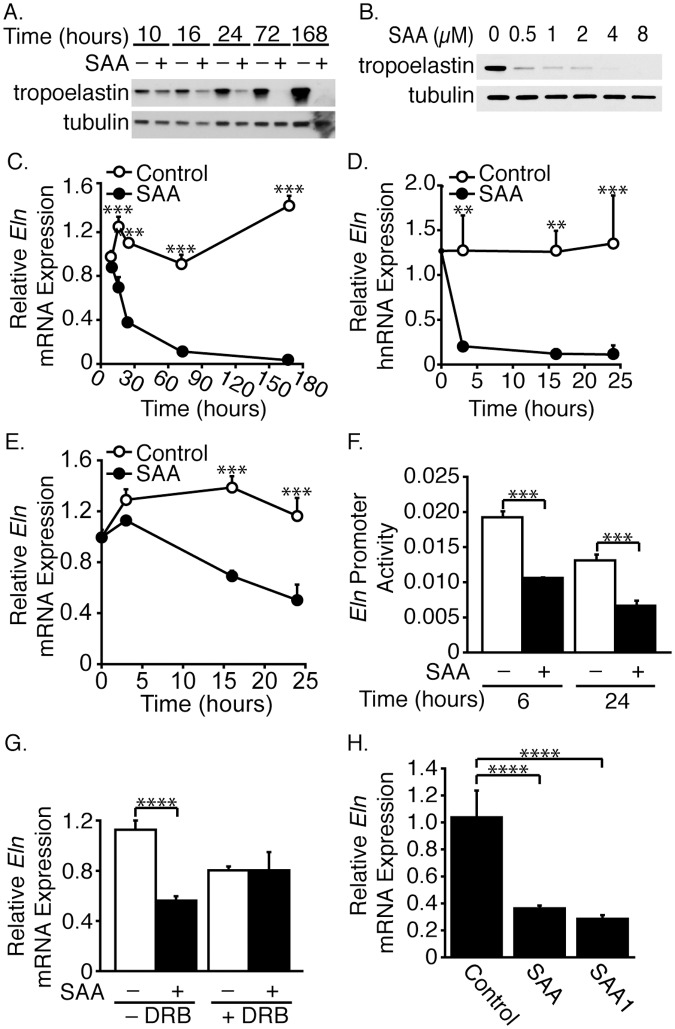
SAA decreases tropoelastin expression. SMCs were treated as in Fig 1A for the indicated time (A) or for 24 hours with the indicated dose (B) and Western blot analysis performed with antibodies directed against tropoelastin and tubulin. SMCs were treated as in Fig 1A (C-E). *Eln* mRNA (C, E) and hnRNA (D) levels are expressed relative to the 10-hour control-treated sample ± SD (n = 3) (C) or 0-hour control-treated sample ± SD (n = 3) (D, E). SMCs were co-transfected with 216-luc and a *Renilla* construct, the latter to normalize for transfection efficiency (F). Cells were treated (or control-treated) with SAA for 6 or 24 hours. Data are expressed as luciferase/*Renilla* ± SD (n = 3). SMCs were treated as in Fig 1D (G). *Eln* mRNA levels are expressed relative to the control-treated (no SAA or DRB) sample ± SD (n = 3). SMCs were treated as in Fig 1F (H). *Eln* mRNA levels are expressed relative to the control-treated sample ± SD (n = 3).

Elastin is synthesized as a monomer (tropoelastin) that is rendered insoluble when covalently cross-linked into the elastic fiber [31]. To determine if increased MMP9 activity may have contributed to decreased tropoelastin expression, Western blot analysis was performed. SAA decreased tropoelastin expression from the earliest harvest time (10 hours) (Fig 2A). By 72 hours, expression was essentially undetectable. Tropoelastin expression continued to increase in control cultures during this time period, consistent with previously reported findings on *Eln* mRNA expression [32]. A dose response demonstrated that even the lowest dose of SAA tested (0.5 μM) decreased tropoelastin expression (Fig 2B).

The large decrease in tropoelastin expression led to consideration of the possibility that the SAA-induced decrease in expression was not due solely to MMP9 activity. Interestingly, SAA had a profound effect on *Eln* mRNA expression with decreases to essentially undetectable levels with time (Fig 2C). Additional studies were performed to determine if the decrease in *Eln* mRNA expression was regulated at the level of gene transcription. As early as 3 hours after adding SAA, there was a dramatic decrease in *Eln* hnRNA that persisted throughout the 24-hour time course, consistent with changes in transcription (Fig 2D). A similar time course showed SAA-induced decreases in *Eln* mRNA (Fig 2E). Transient transfection analyses with an *Eln* promoter construct (216-luc) showed that SAA decreased *Eln* promoter activity at 6- and 24-hour time points (Fig 2F). DRB prevented the SAA-mediated decrease in *Eln* mRNA expression (Fig 2G). It is noteworthy that *Eln* mRNA is very stable as evidenced by the relatively high levels of expression that remained in the presence of DRB.

Comparisons of *Eln* mRNA expression in the presence of the hybrid SAA *vs*. SAA1 revealed that SAA1 was as effective as the hybrid molecule in down-regulating expression (Fig 2H).

### Inhibition of the proteasome and NFκB alter SAA-induced effects

**Fig 3:**
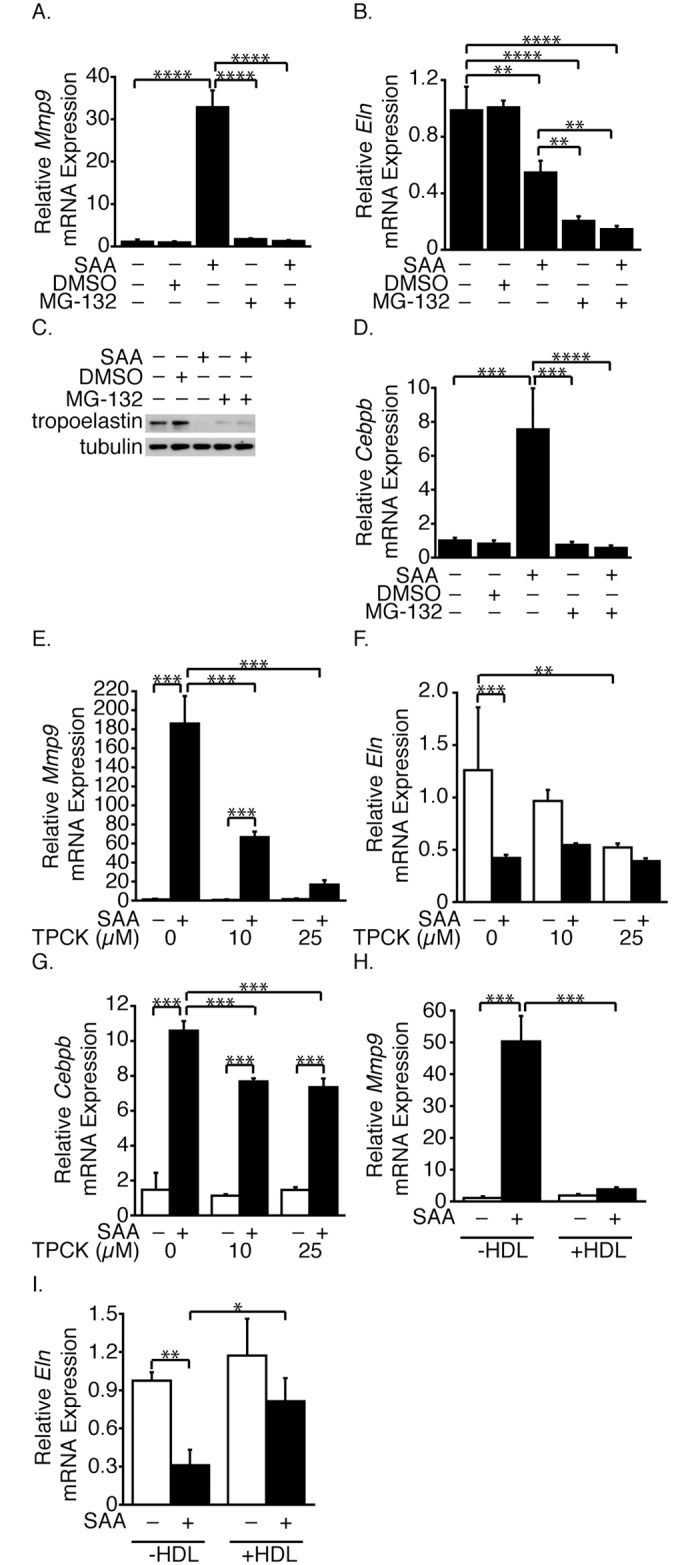
Proteasomes, NFκB and C/EBPβ impact SAA-mediated up-regulation of *Mmp9* mRNA and down-regulation of *Eln* mRNA. SMCs were pretreated with MG-132, then treated (or control-treated) with SAA and incubated for 24 hours (A, B). *Mmp9* (A) and *Eln* (B) mRNA levels are expressed relative to the control-treated (no DMSO, SAA or MG-132) sample ± SD (n = 3). SMCs were treated as in Fig 3A and Western blot analysis performed with antibodies directed against tropoelastin and tubulin (C). SMCs were treated as in Fig 3A (D). *Cebpb* mRNA levels are expressed relative to the control-treated (no DMSO, SAA or MG-132) sample ± SD (n = 3). SMCs were pretreated (or control-treated) with TPCK, then treated (or control-treated) with SAA and incubated for 24 hours (E-G). *Mmp9* (E), *Eln* (F) and *Cebpb* (G) mRNA levels are expressed relative to the control-treated (no DMSO, SAA or TPCK) sample ± SD (n = 3). SMCs were treated (or control-treated) with SAA in the presence or absence of HDL for 24 hours (H, I). *Mmp9* (H) and *Eln* mRNA (I) levels are expressed relative to the control-treated sample ± SD (n = 3).

We previously reported that SAA activates NFκB and up-regulates CCAAT/enhancer binding β (C/EBPβ) expression [21], both regulated by the proteasome [33, 34]. To determine if the proteasome played a role in the SAA-induced changes, cells were pretreated with a proteasome inhibitor, MG-132 [35]. SAA-mediated *Mmp9* mRNA expression was fully inhibited by MG-132 (Fig 3A). SAA alone, MG-132 alone and MG-132 plus SAA decreased both *Eln* mRNA (Fig 3B) and tropoelastin (Fig 3C) expression however, the SAA-induced decrease in *Eln* mRNA was less than that of the MG-132-induced (or MG-132 plus SAA-induced) whereas the effect on tropoelastin was greatest with SAA alone. MG-132 also inhibited the SAA-induced increase in *Cebpb* mRNA expression (Fig 3D). TPCK, an NFκB inhibitor [36], also inhibited the SAA-mediated up-regulation of *Mmp9* mRNA expression (Fig 3E). Baseline *Eln* mRNA expression was inhibited by TPCK however the SAA-mediated down-regulation remained unaffected (Fig 3F). Although a small but significant decrease in the SAA-mediated up-regulation of *Cebpb* mRNA was observed, *Cebpb* mRNA remained elevated in cells treated with SAA and TPCK (Fig 3G). SAA binds HDL [4], which inhibited the SAA-mediated changes in *Mmp9* (Fig 3H) and *Eln* mRNA (Fig 3I).

### Long-term SAA treatment decreases insoluble elastin accumulation

**Fig 4:**
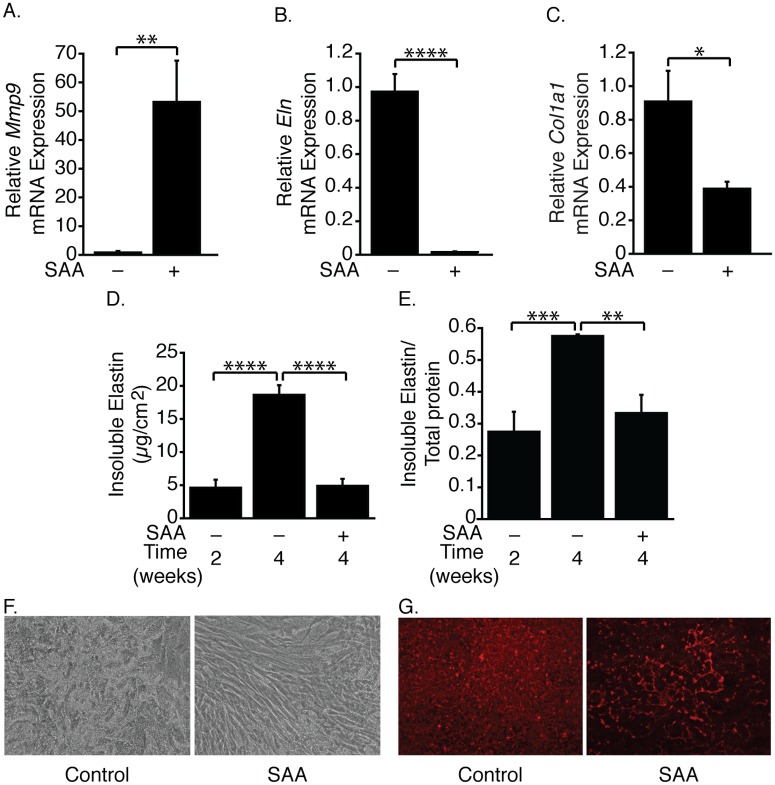
Long-term treatment with SAA decreases insoluble elastin accumulation. SMCs were cultured for 2 weeks, then treated (or control-treated) with SAA for 2 weeks (A-C). *Mmp9* (A), *Eln* (B) and *Col1a1* (C) mRNA levels are expressed relative to the control-treated sample ± SD (n = 3). SMCs were cultured for 2 weeks, then either harvested for baseline measurements or treated (or control-treated) with SAA for 2 weeks (D, E). Insoluble elastin and total protein were determined by amino acid analysis. Data are expressed as insoluble elastin (μg/cm^2^) ± SD (n = 3–4) (D) or insoluble elastin (μg/cm^2^)/insoluble elastin (μg/cm^2^) + hot alkali-soluble protein (μg/cm^2^) ± SD (n = 3–4) (E). SMCs were treated as in Fig 4A (F, G). Cultures were visualized by phase contrast microscopy (F) or stained with Congo red and visualized by fluorescence microscopy (G).

To determine if the SAA-mediated effects on tropoelastin and MMP9 expression were realized in decreased accumulation of insoluble elastin, cells were first maintained for 2 weeks to allow extracellular matrix accumulation and then treated with SAA for 2 weeks. Similar to shorter treatments, SAA increased *Mmp9* mRNA expression (Fig 4A) and decreased *Eln* mRNA expression (Fig 4B). Interestingly, 2 weeks of SAA treatment decreased *Col1a1* mRNA expression (Fig 4C) although this effect was not evident at the earlier time points (data not shown).

Measurements of the hot alkali-insoluble *i*.*e*. insoluble elastin, as well as the hot alkali-soluble protein demonstrated that control cultures accumulated insoluble elastin from 2 to 4 weeks (Fig 4D and 4E). Insoluble elastin in SAA-treated cultures however, was not different from the cultures 2 weeks earlier and was significantly less than that in the control cultures harvested at the same time. In comparison to control-treated cells, phase contrast images revealed reduced extracellular matrix deposition in the SAA-treated cultures (Fig 4F). Decreased insoluble elastin was also observed by Congo red staining (Fig 4G).

### Effects of SAA are IL-1R-independent

**Fig 5:**
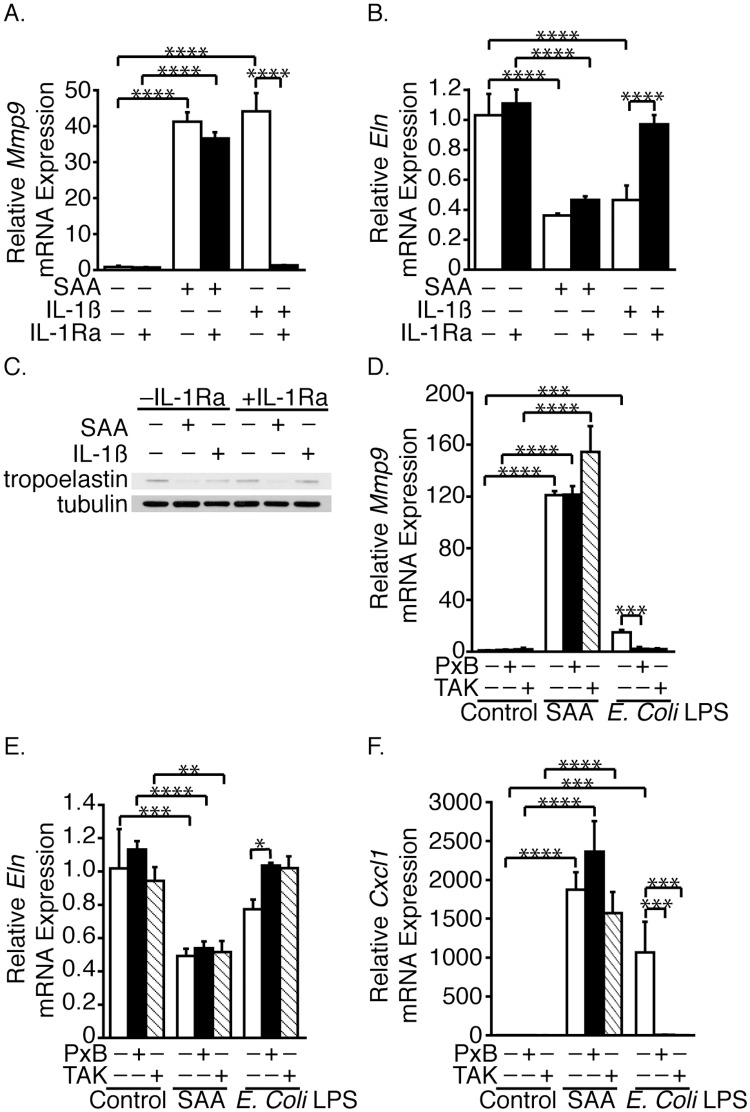
SAA-induced effects are IL-1R- and TLR4-independent. SMCs were pretreated (or control-treated) with IL-1Ra, then treated (or control-treated) with SAA or IL-1β and incubated for 24 hours (A, B). *Mmp9* (A) and *Eln* mRNA (B) levels are expressed relative to the control-treated (no IL-1Ra, SAA or IL-1β) sample ± SD (n = 3). SMCs were treated as in Fig 5A and Western blot analysis performed with antibodies directed against tropoelastin and tubulin (C). SMCs were pretreated (or control-treated) with TAK or PxB, then treated (or control-treated) with SAA or *E*. *Coli* LPS and incubated for 24 hours (D-F). *Mmp9* (D), *Eln* (E) and *Cxcl1* (F) mRNA levels are expressed relative to the control-treated (no PxB, TAK or SAA) sample ± SD (n = 3).

We previously showed that SAA induces SMC IL-1β expression [21]. IL-1β increases SMC MMP9 expression [37] and decreases lung fibroblast elastin expression [24, 38, 39]. Therefore, the possibility that the SAA-induced effects on MMP9 and tropoelastin were IL-1R-mediated was explored using the IL-1Ra. IL-1Ra had no effect on either the SAA-mediated increase in expression of *Mmp9* mRNA (Fig 5A) or the decreases in expression of *Eln* mRNA (Fig 5B) and tropoelastin (Fig 5C). Not unexpectedly, IL-1β increased *Mmp9* expression, decreased *Eln* mRNA and tropoelastin expression and these effects were blocked by the IL-1Ra.

### Effects of SAA are TLR4-independent

To investigate if SAA was functioning as a TLR4 agonist, the effects of PxB [40] and TAK [41] were studied in cells treated with SAA or the TLR4 agonist, *E*. *coli* LPS. *E*. *coli* LPS increased *Mmp9* mRNA expression and this increase was inhibited by both PxB and TAK (Fig 5D). *E*. *coli* LPS had only a small (not statistically significant) effect on *Eln* mRNA expression (Fig 5E). Neither PxB nor TAK affected the SAA-induced changes in *Mmp9* or *Eln* mRNA. The *E*. *coli* LPS preparation used was active, as both *E*. *coli* LPS and SAA induced *Cxcl1* mRNA expression [42, 43]; the *E*. *coli* LPS-mediated, but not the SAA-mediated, increase in *Cxcl1* mRNA expression was blocked by PxB and TAK (Fig 5F).

### TLR2 activation increases Mmp9 mRNA and decreases Eln mRNA expression

**Fig 6:**
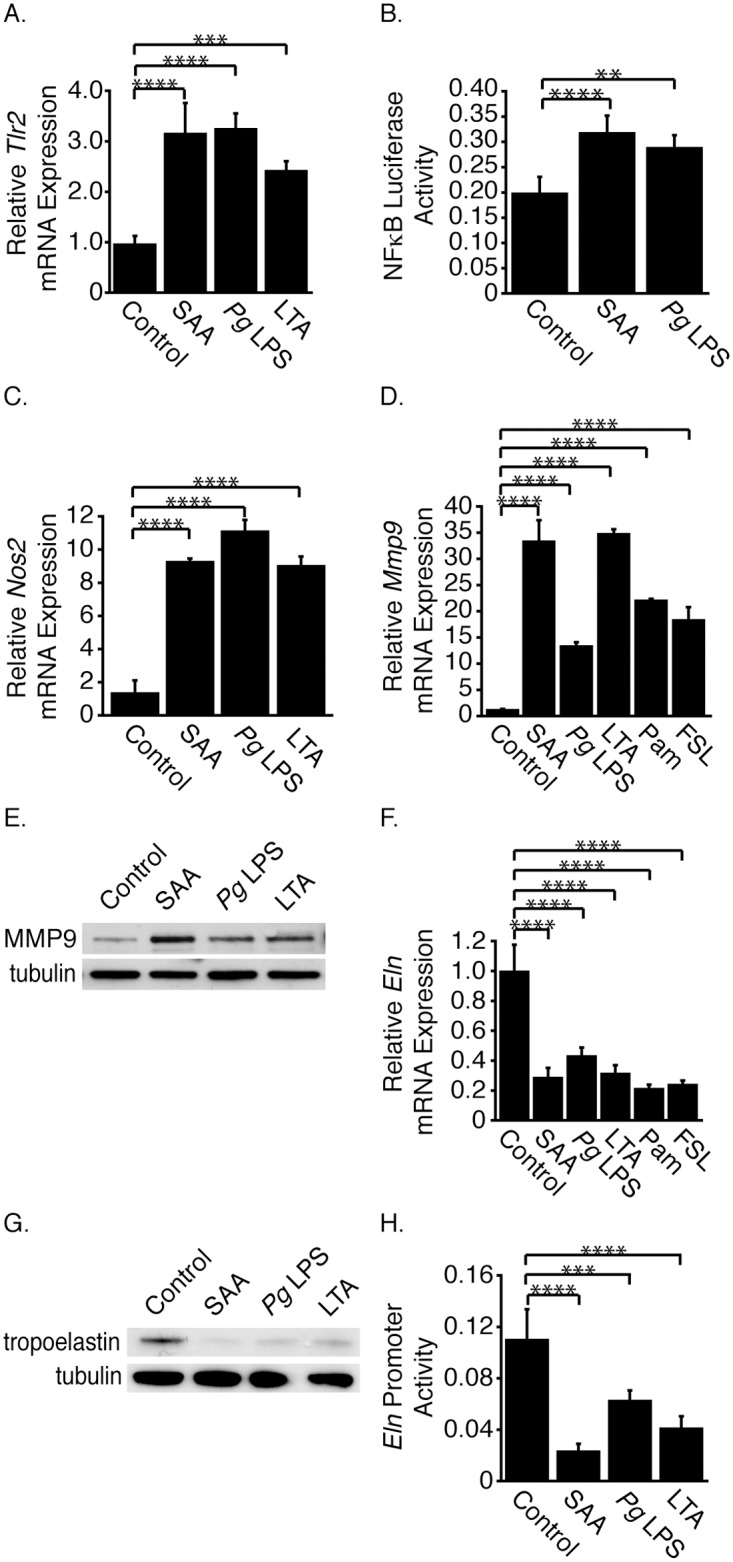
TLR2 activation increases MMP9 expression and decreases tropoelastin expression. SMCs were treated (or control-treated) with SAA, *Pg* LPS or LTA for 24 hours (A). *Tlr2* mRNA levels are expressed relative to the control-treated sample ± SD (n = 3). SMCs were co-transfected with NFκB-luc and a *Renilla* construct, the latter to normalize transfection efficiency (B). Cells were treated (or control-treated) with SAA or *Pg* LPS for 24 hours. Data are expressed as luciferase/*Renilla* ± SD (n = 5). SMCs were treated with SAA, *Pg* LPS, LTA, Pam or FSL as in Fig 6A (C, D). *Nos2* (C) and *Mmp9* (D) mRNA levels are expressed relative to the control-treated sample ± SD (n = 3). SMCs were treated as in Fig 6A and Western blot analysis performed with antibodies directed against MMP9 and tubulin (E). SMCs were treated as in Fig 6D (F). *Eln* mRNA levels are expressed relative to the control-treated sample ± SD (n = 3). SMCs were treated as in Fig 6A and Western blot analysis performed with antibodies directed against tropoelastin and tubulin (G). SMCs were transfected as in Fig 2F (H). Cells were treated (or control-treated) with SAA, *Pg* LPS or LTA for 24 hours. Data are expressed as luciferase/*Renilla* ± SD (n = 5).

To investigate if SAA-activated TLR2 signaling mediated the effects on gene expression, TLR2 expression was first assessed. TLR2 activation up-regulates *Tlr2* gene expression in an autocrine loop [44]. The TLR2 agonists *Pg* LPS and LTA increased *Tlr2* mRNA expression, demonstrating that the receptor was available and the signaling pathway intact (Fig 6A). Interestingly, SAA also activated *Tlr2* mRNA expression. Another known hallmark of TLR stimulation is NFκB [45] activation and as we showed previously, SAA increased NFκB promoter activity [21]; moreover, *Pg* LPS increased NFκB promoter activity (Fig 6B). SAA, *Pg* LPS and LTA increased *Nos2* mRNA expression, another known downstream target of TLR2 [46] (Fig 6C).

*Pg* LPS, LTA, as well as additional TLR2 ligands [Pam and FSL], increased *Mmp9* mRNA expression (Fig 6D). *Pg* LPS and LTA also increased MMP9 protein expression (Fig 6E). Strikingly, these agonists decreased *Eln* mRNA (Fig 6F) and tropoelastin protein expression (Fig 6G). This effect was at the transcriptional level as evidenced by decreased *Eln* promoter activity in *Pg* LPS- and LTA-treated cells (Fig 6H).

### SAA effects are TLR2-dependent

**Fig 7:**
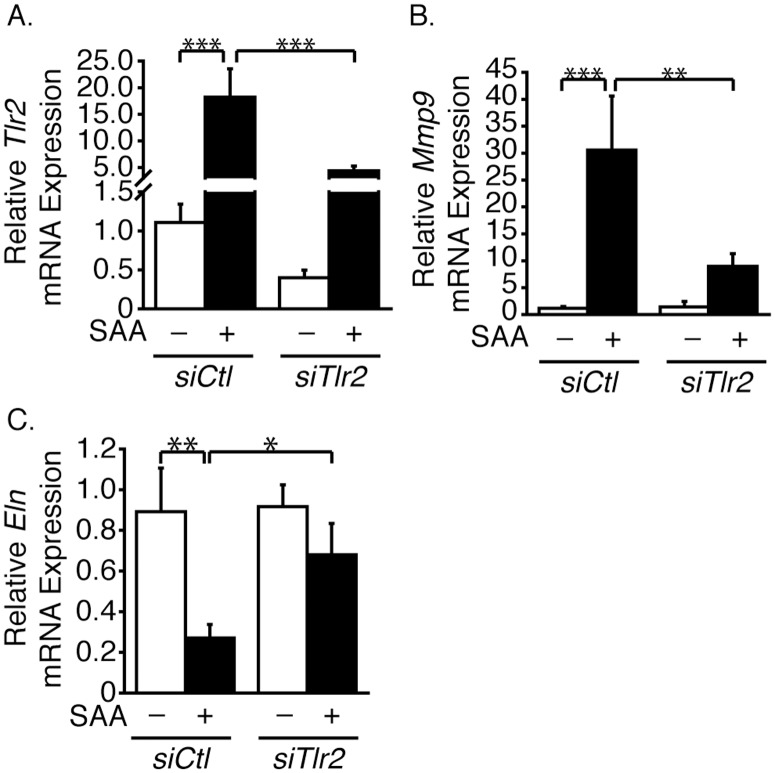
SAA up-regulates *Mmp9* mRNA expression and down-regulates *Eln* mRNA expression via TLR2. SMCs pretreated with *siTlr2* or a control *(siCtl*) were treated (+) or control-treated (-) with SAA for 24 hours. *Tlr2* (A), *Mmp9* (B) and *Eln* (C) mRNA levels are expressed relative to the control-treated (*siCtl*, no SAA) sample ± SD (n = 3).

Small interfering RNA targeting rat TLR2 (*siTlr2*) was used to knock down TLR2 expression. Knockdown efficiency was 64% (Fig 7A). As shown above, SAA up-regulated *Tlr2* mRNA expression in *siCtl*-treated cells and not unexpectedly, since the knockdown was not complete, SAA up-regulated *Tlr2* mRNA expression in the *siTlr2*-treated cells as well. Compared to *siCtl*-treated cells, there was a decrease in the effect of SAA on *Mmp9* mRNA (Fig 7B). Likewise, *siTlr2* invoked a significant reversal of the effect of SAA on the down-regulation of *Eln* mRNA expression (Fig 7C).

## Discussion

### Extracellular matrix in the vasculature; SAA and TLR2

The physiological function of SAA is not well understood, and both pro- and anti-atherogenic effects have been reported [3]. Undoubtedly, the physiologic/pathologic context is critical, particularly in light of a number of reports showing that SAA is a ligand for a variety of receptors such that cell type and receptor availability are certain to contribute to distinct SAA-mediated outcomes. Relevant to this report, Cheng et *al*. demonstrated binding of SAA and TLR2, concluding that SAA is a TLR2 ligand [10]. These studies show SAA-induced changes in SMC MMP9 and tropoelastin expression. The decrease in elastin was evident at the level of mRNA, protein expression and insoluble accumulation. As both tropoelastin and insoluble elastin are MMP9 substrates [47, 48], the SAA-mediated increase in MMP9 likely contributed to the decreased insoluble elastin. Effects were IL-1R-independent, TLR4-independent and TLR2-dependent. Moreover, as relevance of the hybrid recombinant human SAA has come into question [49], this study showed that SAA1 was at least as effective as the hybrid product. Interestingly, TLR2 activation by bacterial components and synthetic TLR2 agonists increased MMP9 and decreased tropoelastin expression.

Little is known about the role of SMC TLR2; Lee et *al*. showed that TLR2 promotes SMC migration [50] and the findings in this report indicate that TLR2 activation impacts both vascular elastin synthesis and MMP9-mediated elastin degradation. The influence of vascular elastin levels on atherosclerosis remains unclear. In addition to providing resilience/elasticity to tissues [51], elastin limits SMC proliferation and migration [52, 53]. Exogenous elastin reduces neointima formation in a porcine restenosis model [53]. MMP9 contributes to lesion formation [54] and it increases SMC migration and replication [55, 56]. Elastin degradation by MMP9 also generates elastin peptides, which may impact atherosclerosis and abdominal aortic aneurysms [57]. Protective effects of MMP9 on atherosclerosis have also been reported [58]. Studies of a transgenic MMP9-expressing mouse model suggest that MMP9 increases collagen deposition in atherosclerosis and could thus contribute to lesion stability [59].

### NFκB and C/EBPß in SAA-mediated effects

IL-1ß-mediated *Eln* gene down-regulation in rat lung fibroblasts results from NFκB activation [24]. Moreover, NFκB activates C/EBPß expression, thereby down-regulating *Eln* expression [39]. We showed that SAA induces p65 translocation to the nucleus and up-regulates C/EBPß [21]. In rat lung fibroblasts, proteasome inhibition down-regulates *Eln* transcription [60] and as these authors found, SMC *Cebpb* mRNA was not affected by MG-132 in this study; the post-transcriptional accumulation of C/EBPß these authors reported is the likely mechanism for the greater decrease in *Eln* mRNA in MG-132-treated cells compared to SAA-treated cells. Tropoelastin expression in SAA-treated cells was lower than in MG-132-treated cells however, providing further evidence that the up-regulation of MMP9 expression in SAA-treated cells (but not MG-132-treated cells) contributed to decreased tropoelastin. MMP9 expression is NFκB-regulated [61] and these data support a role for NFκB in SAA-mediated MMP9 expression as well as baseline tropoelastin expression. Moreover, the data suggest that C/EBPß maintains the SAA-mediated down-regulation of *Eln* mRNA in the presence of the NFκB inhibitor. C/EBP activates the *COL1A1* promoter [62] but NFκB interferes with Sp-1-induced transcription of *COL1A1* [63]. This likely explains why collagen expression was down-regulated only with prolonged exposure to SAA.

### TLR2-dependent effects on elastin accumulation

Activation of the IL-1R decreases lung fibroblast *Eln* gene expression [24, 38, 39]. These data extend the findings to SMCs. We showed that SAA induces IL-1β expression [21], but the SAA-mediated effects on *Eln* and *Mmp9* mRNA were IL-1R-independent. TLR2 and TLR4 have been implicated as SAA receptors [10, 13], therefore the possibility that SAA activated these receptors to induce the SMC-mediated changes was explored. It was also important to rule out endotoxin-associated effects on TLR4 [64] since endotoxin contamination of recombinant proteins is inevitable. *E*. *coli* LPS at a concentration greater than 100-times higher than the small amount of endotoxin potentially contaminating the SAA (0.01 ng *E*. *Coli* LPS/μg SAA) had no effect on *Eln* mRNA expression, and although it increased *Mmp9* mRNA expression to some extent, this was prevented by the TLR4 inhibitors, which had no effect on the SAA-induced changes in *Mmp9* and *Eln* mRNA. Thus, endotoxin contamination of the SAA was not responsible for the effects on MMP9 and tropoelastin expression and the SAA-induced effects were TLR4-independent.

TLR2 activation has been implicated in atherosclerosis [14] but the role of TLRs on the vulnerability of plaque/remodeling of the lesion remains uncertain [65]. Of great interest is that SMC TLR2 activation down-regulated tropoelastin expression. The results showed the novel down-regulation of tropoelastin expression by not only SAA but also by other TLR2 agonists (bacterial components and synthetic ligands), dissimilar in structure to SAA. Much of the work on TLR2 has evaluated its function in cells classically associated with the immune system; in addition to a report demonstrating that TLR2 activation induces SMC migration [50], this work extends TLR2 function to SMCs. Importantly, the study provides a novel mechanism for the role of TLR2 in atherosclerosis, offering a link between infection with periodontal pathogens *e*.*g*. *P*. *gingivalis*, respiratory pathogens *e*.*g*. *Chlamydia pneumonia* and changes in vascular extracellular matrix consistent with atherosclerosis [17, 18].

In conclusion, the SAA and TLR2-mediated loss of accumulation of extracellular matrix may impact the vascular processes of SMC-mediated plaque remodeling and rupture, leading to myocardial infarction and stroke.
